# Supplementary material for: TLR7 Promotes Acute Inflammatory-Driven Lung Dysfunction in Influenza-Infected Mice but Prevents Late Airway Hyperresponsiveness
Source: Int J Mol Sci. 2024 Dec 21;25(24):13699. doi: 10.3390/ijms252413699 (PMC11678220; doi:10.3390/ijms252413699)
Supplement: Supplementary file 1 [file ijms-25-13699-s001.zip › ijms-3364504-supplementary.pdf]

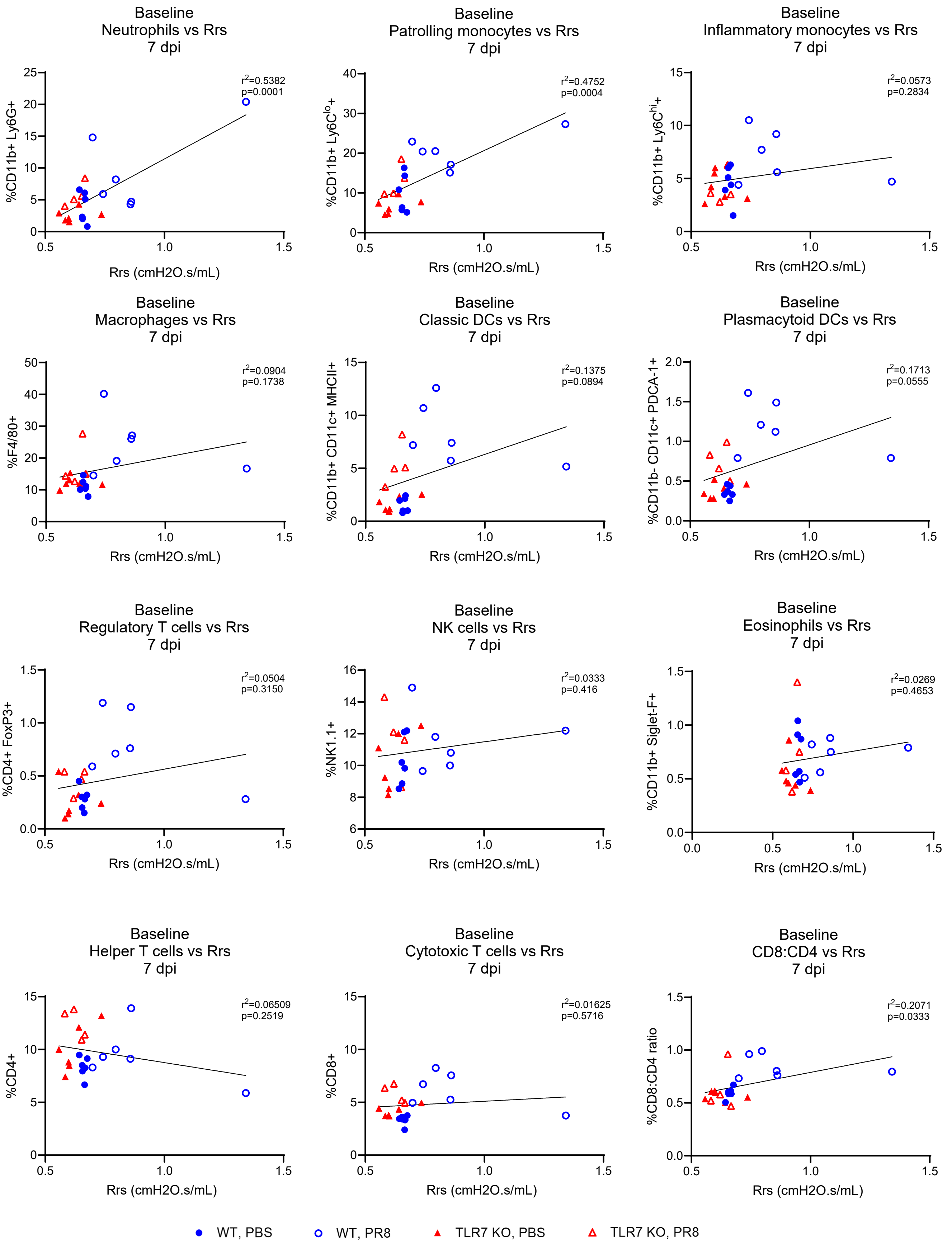

Figure S1. Correlation of baseline respiratory resistance with lung immune cells at 7 dpi. Simple linear regression tests were performed on C57Bl/6 or TLR7 KO mice that were infected with PR8 (50 PFUs) or PBS (control) after 7 dpi. Comparisons were made for lung infiltrating immune cell types with the degree of respiratory system resistance (Rrs) for each mouse at baseline. Statistical analysis was conducted using a simple linear regression test (n=4-6).

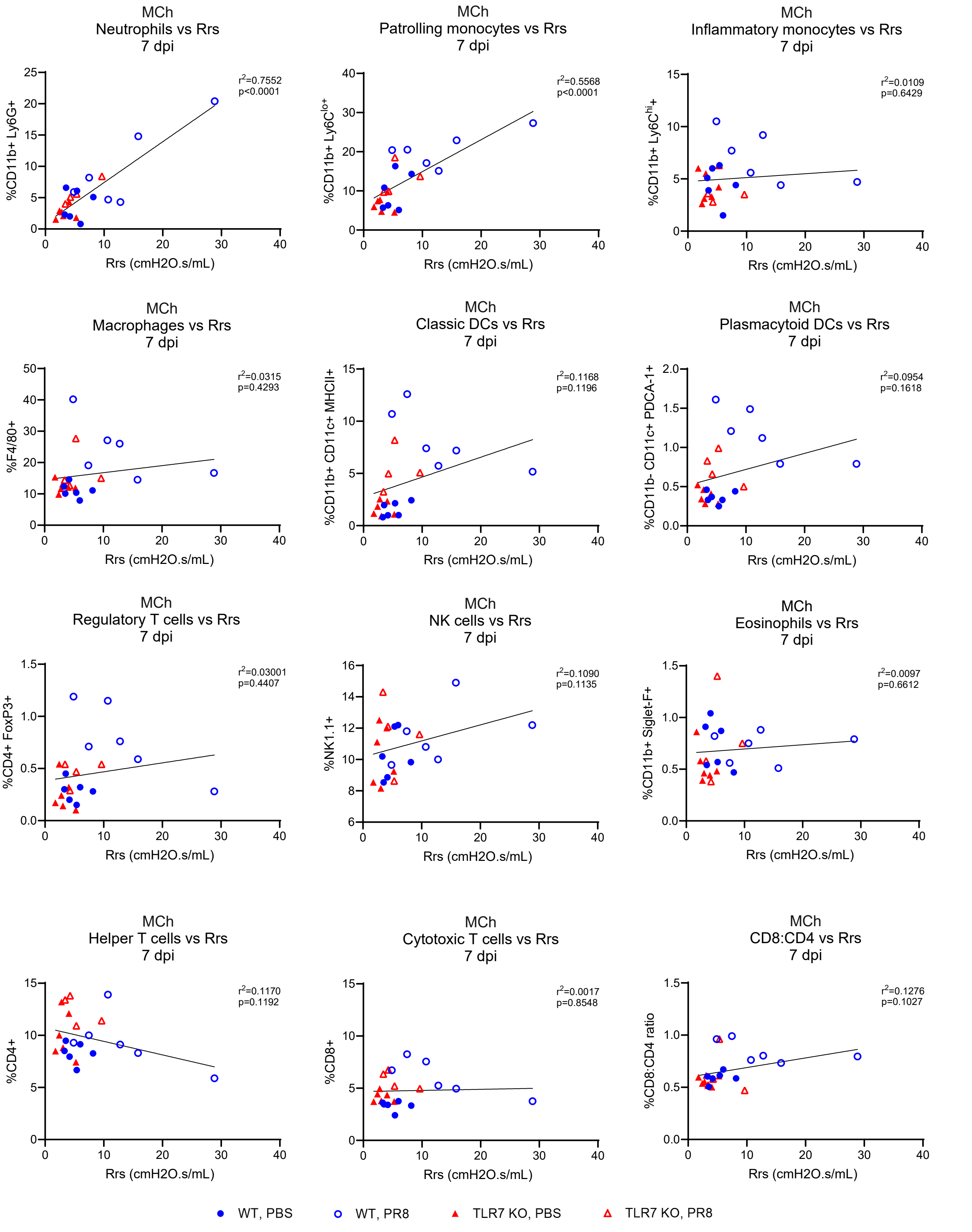

Figure S2. Correlation of MCh-induced respiratory resistance with lung immune cells at 7 dpi. Simple linear regression tests were performed on C57Bl/6 or TLR7 KO mice that were infected with PR8 (50 PFUs) or PBS (control) after 7 dpi. Comparisons were made for lung infiltrating immune cell types with the degree of respiratory system resistance (Rrs) for each mouse after challenge with 100 mg/ml MCh. Statistical analysis was conducted using a simple linear regression test (n=4-6).

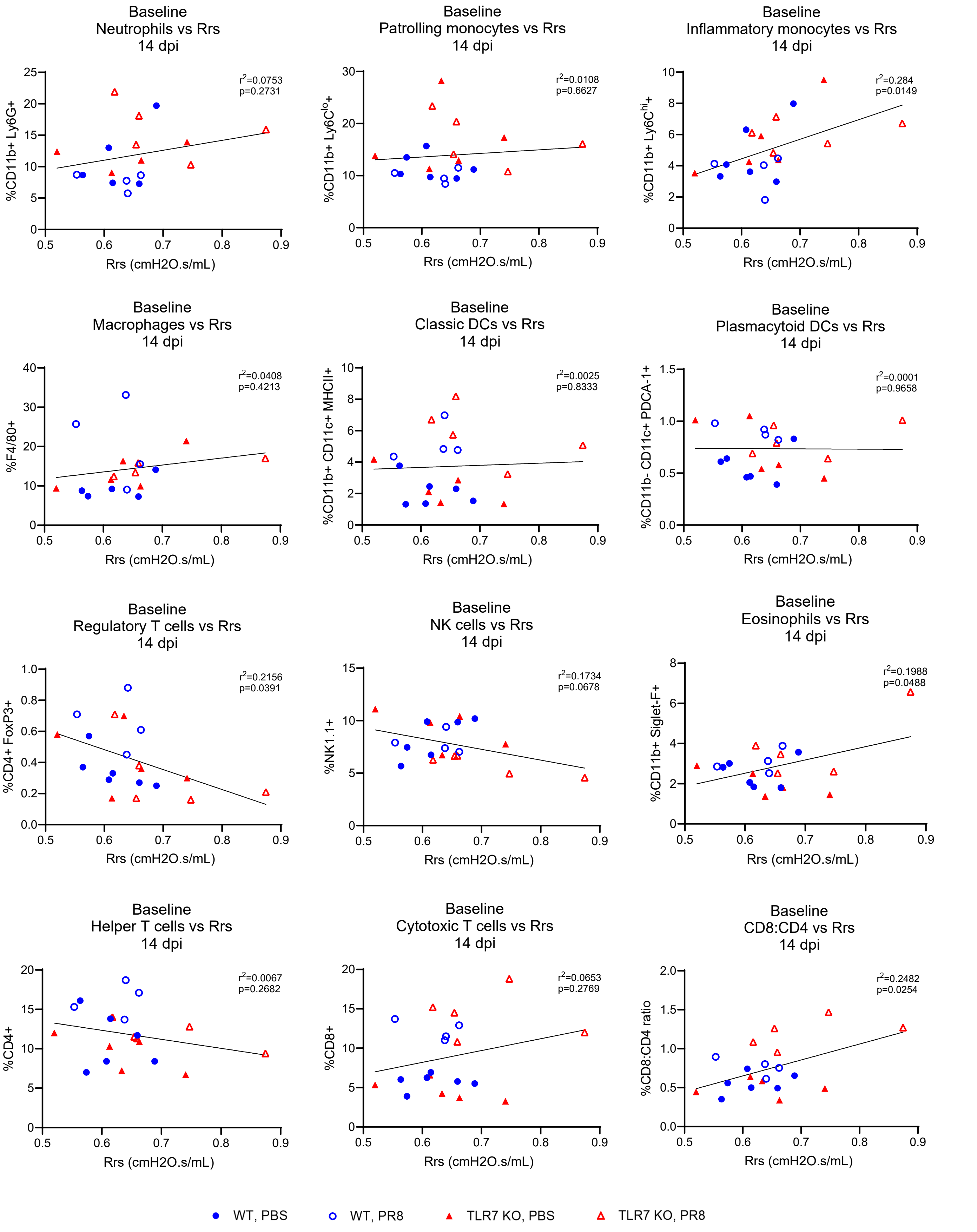

Figure S3. Correlation of baseline respiratory resistance with lung immune cells at 14 dpi. Simple linear regression tests were performed on C57Bl/6 or TLR7 KO mice that were infected with PR8 (50 PFUs) or PBS (control) after 14 dpi. Comparisons were made for lung infiltrating immune cell types with the degree of respiratory system resistance (Rrs) for each mouse at baseline. Statistical analysis was conducted using a simple linear regression test (n=4-6).

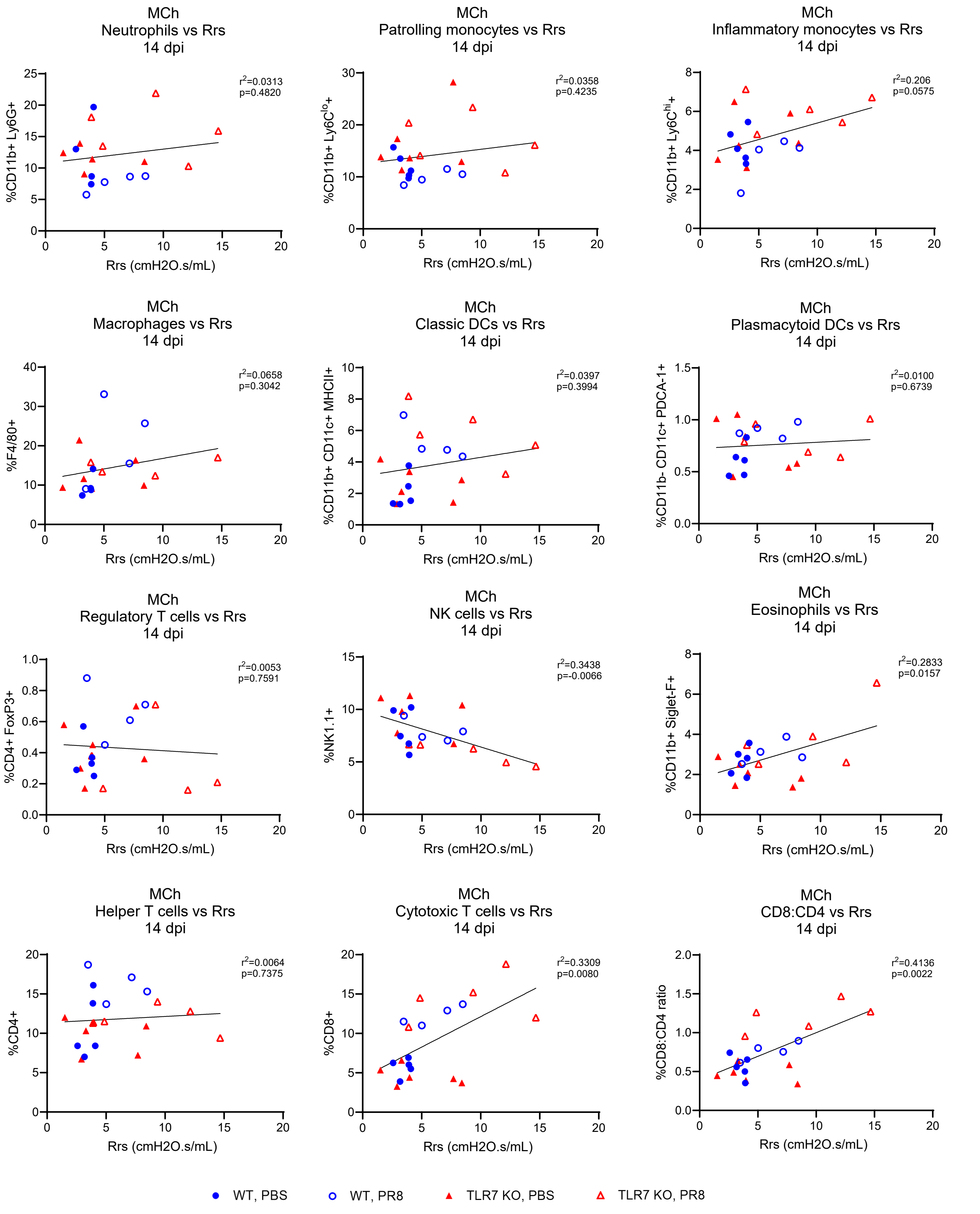

Figure S4. Correlation of MCh-induced respiratory resistance with lung immune cells at 14 dpi. Simple linear regression tests were performed on C57Bl/6 or TLR7 KO mice that were infected with PR8 (50 PFUs) or PBS (control) after 14 dpi. Comparisons were made for lung infiltrating immune cell types with the degree of respiratory system resistance (Rrs) for each mouse after challenge with 100 mg/ml MCh. Statistical analysis was conducted using a simple linear regression test (n=4-6).

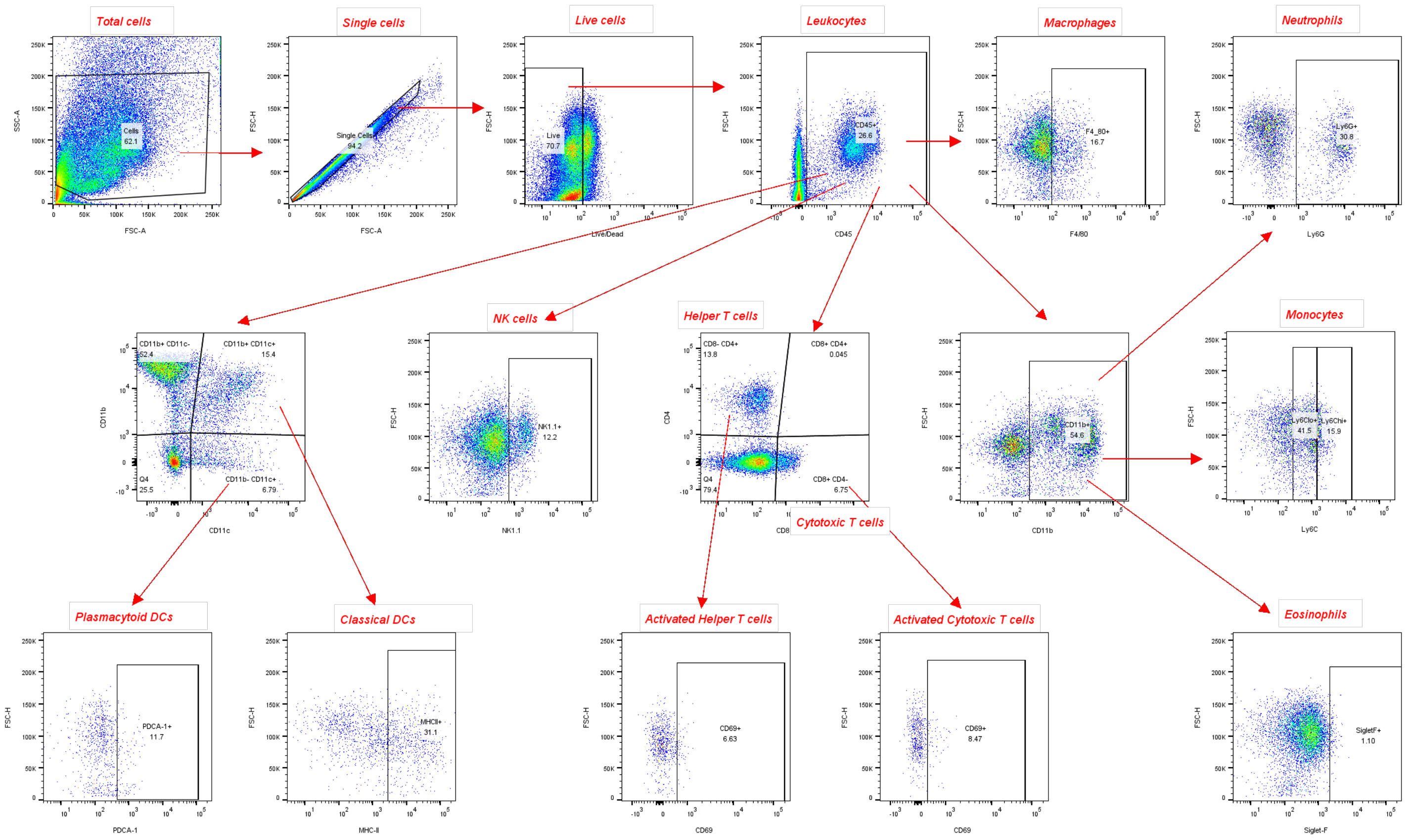

Figure S5. Flow cytometry gating strategy.

Table S1. Summary of linear regression analysis of Rn at baseline and MCh-induced with lung immune cell populations across PR8 infection.

Lung Correlation with Rn	7 dpi				14 dpi			
	Baseline		Methacholine		Baseline		Methacholine	
Cell type	P-value	R^2 value	P-value	R^2 value	P-value	R^2 value	P-value	R^2 value
Macrophages	0.4308	0.0369	0.6784	0.0117	-0.2331	0.0993	0. 6207	0.0019
Inflammatory monocytes	0.3546	0.0505	0.8355	0.0029	0.678	0.011	0. 0205	0.3029
Patrolling monocytes	0.6766	0.0105	0.0448	0.2421	0.3502	0.0547	0.178	0.1175
Neutrophils	0.9245	0.0092	0.0252	0.2918	0.593	0.021	0.0714	0.2137
Classic DCs	0.7527	0.0056	0.0066	0.3987	-0.8711	0.0017	0.4417	0.04
Plasmacytoid DCs	0.2128	0.0897	0.1258	0.1491	-0.3787	0.0487	0.1824	0.1153
Eosinophils	0.8597	0.0018	0.6583	0.134	0.4477	0.0365	0.0387	0.2551
NK cells	0.8823	0.0013	0.1252	0.1495	0.9997	0.0001	-0.008	0.384
Regulatory T cells	0.2575	0.0747	0.1648	0.1245	0.7992	0.0041	-0.0328	0.2629
Helper T cells	0.578	0.0186	0.2546	0.0856	0.9929	0.0005	-0.3549	0.0573
Cytotoxic T cells	0.3386	0.0539	0.0099	0.3677	-0.6332	0.0146	0.0903	0.1793
CD8:CD4 ratio	0.5711	0.0193	0.1401	0.1393	-0.6254	0.0152	0.0015	0.498
